# Screening of Pig-Derived Zearalenone-Degrading Bacteria through the Zearalenone Challenge Model, and Their Degradation Characteristics

**DOI:** 10.3390/toxins14030224

**Published:** 2022-03-18

**Authors:** Xue Yang, Feng Li, Hangyi Ning, Wei Zhang, Dongyan Niu, Zhuo Shi, Sa Chai, Anshan Shan

**Affiliations:** 1 Institute of Animal Nutrition, College of Animal Science and Technology, Northeast Agricultural University, Harbin 150030, China; yangxue01@caas.cn (X.Y.); ninghangyi@163.com (H.N.); weizhang@henau.edu.cn (W.Z.); s210501041@163.com (Z.S.); chaisa1203@163.com (S.C.); asshan@neau.edu.cn (A.S.); 2 Institute of Special Animal and Plant Sciences, Chinese Academy of Agricultural Sciences, Changchun 130112, China; 3 Department of Animal Nutrition and Feed Science, College of Animal Science and Technology, Henan Agricultural University, Zhengzhou 450046, China; 4 Department of Ecosystem and Public Health, Faculty of Veterinary Medicine, University of Calgary, Calgary, AB T2N 4Z6, Canada; dongyan.niu@ucalgary.ca

**Keywords:** zearalenone, *Proteus mirabilis*, *Bacillus subtilis*, pig-derived zearalenone-degrading strains, zearalenone challenge pig model

## Abstract

Zearalenone (ZEN) is widely found in food and feed. Its cytotoxicity, reproductive toxicity, genetic toxicity, immunotoxicity and hepatorenal toxicity have serious impacts on human and animal health. In order to help animals avoid ZEN poisoning in feed, ZEN-degrading bacterial strains were screened from fecal samples through a zearalenone challenge pig model, and their degradation characteristics were researched. Through the optimization of parameters such as the culture time, pH value, temperature, and strain concentration, the optimal conditions for the ZEN-degrading ability of these strains were preliminarily determined, and the active site of the ZEN degradation was explored. In this study, three strains (SY-3, SY-14, SY-20) with high ZEN degradation capacities were obtained. SY-3 was identified as *Proteus mirabilis*, and its main degrading component was the supernatant. SY-14 and SY-20 were identified as *Bacillus subtilis*. Their main degrading components were the intracellular fluid of SY-14, and the intracellular fluid and cell wall of SY-20. The above results showed that the ZEN challenge model was an effective way to screen ZEN-degrading bacteria.

## 1. Introduction

Mycotoxin contamination has become an increasing concern globally. Zearalenone (ZEN) is one of the most widely used mycotoxins, and is a secondary metabolite produced by several *Fusarium* species [1]. The major mycotoxin-producing molds include *Fusarium graminearum*, *Fusarium tricinctum*, *Fusarium culmorum*, *Fusarium equiseti*, *Fusarium sernitectum* and *Fusarium solani* [2]. ZEN was first isolated from corn contaminated with *Fusarium graminearum* in 1962 [3]. The complete chemical structure of ZEN was determined by Urry et al. in 1966 [4]. A survey on the occurrence of mycotoxin contamination in feed, feedstuffs and cereals showed that the majority of countries have different degrees of ZEN contamination [1]. Another survey on the occurrence of ZEN contamination in 3507 feed samples from 22 provinces of China during 2018–2020 showed that the detection rate of ZEN was nearly 96.4%, and 0.5% of feed ingredients with ZEN exceeded China’s safety standards [5].

ZEN is an estrogen-like mycotoxin that results in a decline in poultry and livestock production, and causes damage to human health and serious economic losses. Obremski et al. [6] reported that apoptoso-like changes in granule cells and ovarian follicle atresia were noted in sexually immature gilts fed ZEN-contaminated diets. Zhang et al. [7] reported that the ingestion of a diet contaminated with ZEN for pregnant female Sprague-Dawley rats during early gestation caused a loss of maternal reproductive capability and delayed fetal development.

It is important to find safe and efficient methods to eliminate or minimize ZEN. Traditionally, physical and chemical methods are usually used to eliminate or minimize ZEN. Physical treatments include high-temperature treatment [8], adsorption [9,10], and irradiation [11]. Chemical treatments include sodium carbonate soaking [12], ozone treatment [13,14] and hydrogen peroxide [15]. Some of the physical and chemical methods presently used are successful, but these methods have some limitations. These methods cause high costs and the loss of important nutrients.

Therefore, the development of a more effective method is needed in order to control ZEN contamination. At present, the biodegradation of ZEN has become one of the most popular topics for research and development. Many studies on the biodegradation of ZEN have been reported. The strain *Rhodococcus pyridinivorans* K408 can degrade ZEN, and the degradation rate is 87.21% [16]. The strain *Aspergillus niger* FS10 was isolated from fermented Chinese soybeans and can degrade ZEN, and the degradation rate is 89.56% [17]. Three strains of *Lactobacillus* plantarum isolated from fermented food have the ability to degrade ZEN [18]. Two strains of *Pseudomonas* isolated from soil can degrade ZEN [19]. ZEN was also found to be degraded by the supernatant of FS10 and absorbed by FS10 cells [17]. ZEN was also found to be absorbed by *Bacillus subtilis* 168, and *Bacillus natto* CICC 24640 was inactivated and treated with acid [20].

At present, several studies on degradation by microorganisms have been reported, and the strains in these studies were isolated mainly from soil or fermented food. A few strains were isolated from animals. Therefore, host specificity is essential in order to achieve good adaptation. A strain of lactic acid bacteria was isolated from pig rectal swabs that could minimize ZEN [21]. The strain of the *Bacillus subtilis* ANSB01G isolates taken from normal broiler intestinal chyme could reduce ZEN by 88.65% [22].

For all of these reasons, the purpose of the present work was to study the ZEN degradation capacity of strains isolated from feces from the ZEN toxicity test of pigs. The strain SY-3 was isolated and identified. In addition, the effects of the culture conditions and active components on the ability of SY-3 to degrade ZEN were evaluated.

## 2. Results

### 2.1. Isolation of ZEN-Degrading Bacteria

**Figure 1:**
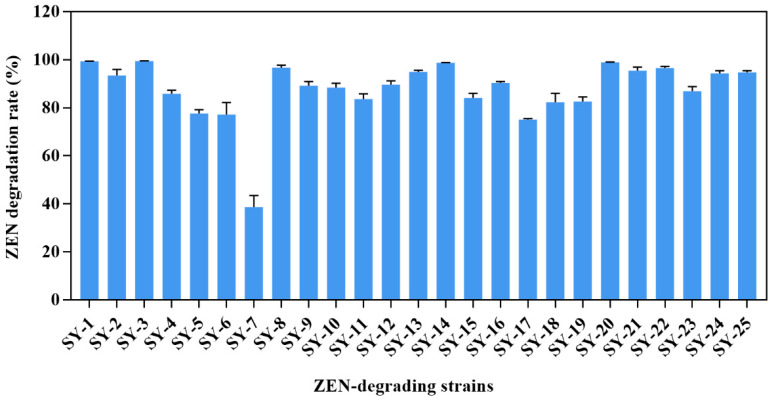
The ZEN degradation rate in Luria–Bertani (LB) by different strains. The different values of the columns are significantly different (*p* < 0.05).

After successive isolation, 25 strains were isolated from the feces of pigs, and the percentage of ZEN degradation exceeded 10%. The twenty-five strains were named SY-1 to SY-25 (Figure 1). In particular, strains SY-3, SY-14 and SY-20 showed higher degradation activities, with ZEN reduction percentages of 99.48%, 98.78% and 98.97%, respectively.

### 2.2. Morphological, Physiological and Biochemical Characterization, and Phylogenetic Analysis

**Figure 2:**
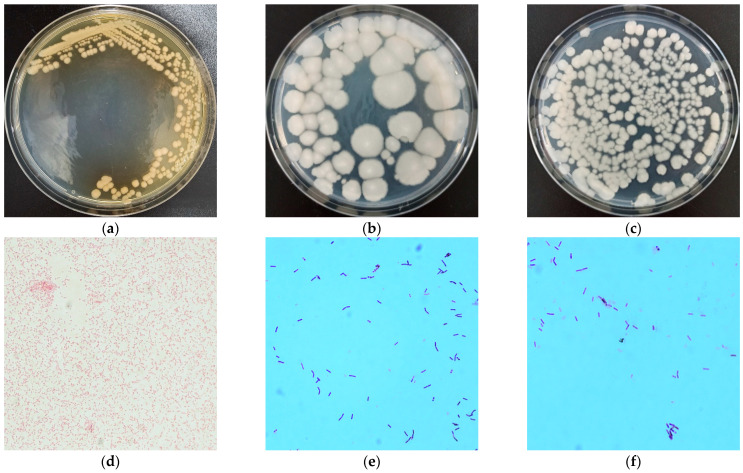
Colony characteristics of the strains: (**a**) SY-3, (**b**) SY-14, and (**c**) SY-20. The cell morphology of the strains: (**d**) SY-3, (**e**) SY-14, and (**f**) SY-20.

The colonies of SY-3 were faint yellow, convex, and irregularly circular, with non-smooth edges on LB agar (Figure 2a). SY-14 formed visible colonies on LB plates; the colonies were irregular, ivory-white, and opaque, with non-smooth edges (Figure 2b). SY-20 exhibited a rough edge that was irregular, ivory-white, and opaque on LB plates (Figure 2c). SY-3 was a Gram-negative bacterium with a short rod shape (Figure 2d). SY-14 was a rod-shaped and Gram-positive bacterium (Figure 2e). SY-20 belonged to Gram-positive, rod-shaped bacteria (Figure 2f). The biochemical characteristics of SY-3, SY-14 and SY-20 are listed in Table 1.

**Figure 3:**
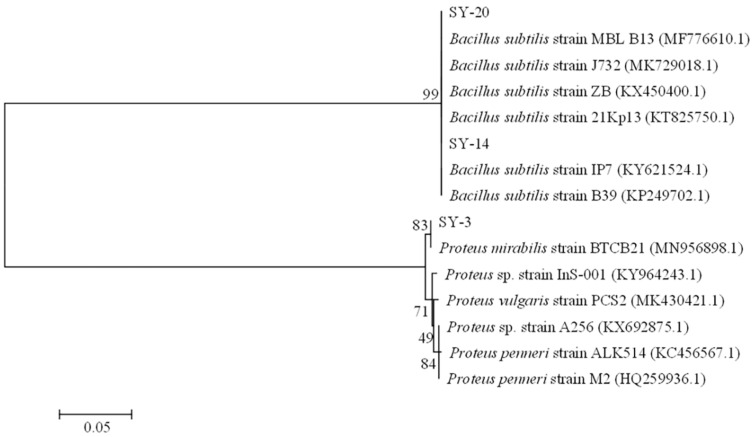
The phylogenetic tree of strains SY-3, SY-14 and SY-20.

The 16S rDNA gene sequences obtained were analyzed using a BLAST search in the NCBI database (https://www.ncbi.nlm.nih.gov/, accessed on 5 January 2020). Strain SY-3 was related to *Proteus mirabilis*, with 100% identity (GenBank accession number: MZ348886). Strains SY-14 and SY-20 were related to *Bacillus subtilis*, with 100% identity. The phylogenetic tree was constructed by the MEGA 6.0 program [23] using neighbor-joining to illustrate the relative position of SY-3 and other *Proteus mirabilis*, SY-14, SY-20, and other *Bacillus subtilis* (Figure 3).

### 2.3. Growth Curves of ZEN-Degrading Strains

**Figure 4:**
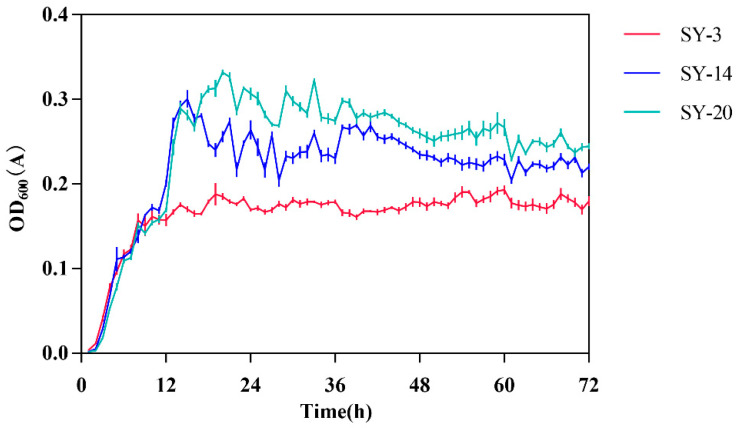
The growth curve of SY-3, SY-14 and SY-20.

The growth curves of three strains were shown in Figure 4. The growth curve of SY-3 showed that 1–8 h was an exponential phase. SY-3 grew slowly after 8 h, and a stationary phase appeared. In the SY-14 culture, 2–15 h was an exponential phase, and a stationary phase appeared after 15 h. The growth curve of SY-20 during 2–17 h was an exponential phase, and a stationary phase appeared after 17 h.

### 2.4. Effect of the Culture Conditions on the Degradation of ZEN

**Figure 5:**
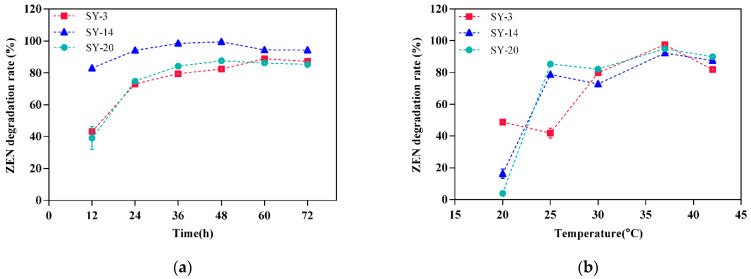

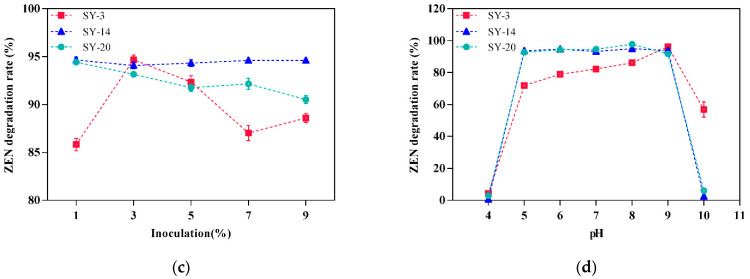
Effect of the culture conditions for strains SY-3, SY-14 and SY-20 on ZEN degradation: (**a**) time, (**b**) temperature, (**c**) inoculation, and (**d**) pH.

The effects of different factors on the ZEN degradation activity in the cultures are shown in Figure 5. ZEN was degraded by SY-3, SY-14 and SY-20 at all of the incubation times used in this study (Figure 5a). The SY-3 degradation rate increased with increasing times from 0 to 60 h, and became stable from 60 to 72 h. There was no significant difference in the degradation time of ZEN between 60 and 72 h (*p* > 0.05); as a result, 60 h was the best culture time of *Proteus mirabilis* SY-3. The SY-14 degradation rate increased with increasing time from 0 to 36 h, and became stable from 36 to 72 h. There was no significant difference in the degradation time of ZEN between 36 and 48 h (*p* > 0.05); therefore, 36 h was the best culture time for *Bacillus subtilis* SY-14. SY-20’s degradation rate increased with increasing time from 0 to 36 h, and became stable from 36 to 72 h. There was no significant difference in the degradation time of ZEN between 36 and 72 h (*p* > 0.05), such that 36 h was the best culture time for *Bacillus subtilis* SY-20.

The degradation of ZEN by SY-3, SY-14 and SY-20 was dependent on the temperature of the bacteria in the medium (Figure 5b). The SY-3 strain, SY-14 strain, and SY-20 strain showed higher degradation rates at 37 °C, with percentages of 97.55%, 92.23% and 94.93% (*p* < 0.05).

ZEN was degraded by SY-3, SY-14 and SY-20 at all of the incubation inoculations used in this study (Figure 5c). The strain SY-3 showed a higher ZEN degradation rate at 3% (*p* < 0.05). There was no significant difference in the incubation inoculations of SY-14 between 1 and 9% (*p* > 0.05). The strain SY-20 showed a higher degradation rate at 1% (*p* < 0.05).

The degradation of ZEN showed little sensitivity to the pH value (Figure 5d). SY-3, SY-14 and SY-20 had higher degradation rates at pH values from 5 to 9 than at pH values of 4 and 10. An increase in the pH from 4 to 9 increased the degradation rate of SY-3, and reached the maximum degradation rate at 9 (*p* < 0.05). There was no significant difference in the degradation capability of SY-14 between pH 5 and pH 9 (*p* > 0.05). SY-20 resulted in the highest degradation rate of 97.76% when cultured at pH 8 (*p* > 0.05).

### 2.5. Determination of the Degradation Ability of Strain Components in ZEN

**Figure 6:**
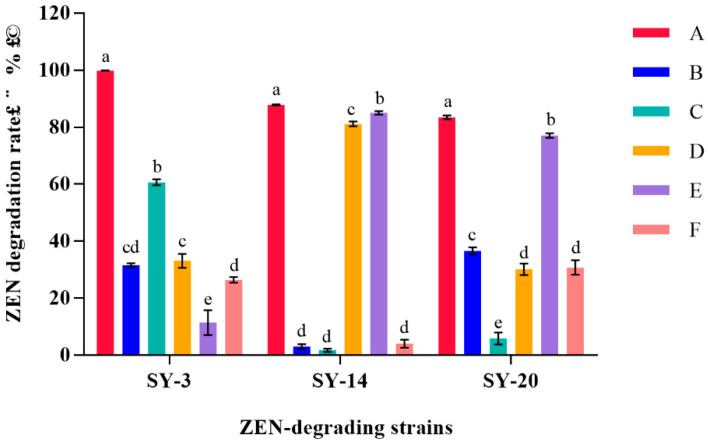
ZEN degradation rate of each component of strain SY-3, SY-14 and SY-20. A, live bacteria; B, inactivated bacteria; C, supernatant; D, cell pellets; E, intracellular fluid; F, cell wall. Different lowercase letters mean a significant difference (*p* < 0.05), and the same letters mean no significant difference (*p* > 0.05).

From the results listed above, we know that the three strains can degrade ZEN with high efficiency. In order to confirm the components for the degradation of ZEN, the three strains were divided into different parts and cultured with 5 μg/mL ZEN for 48 h. After cultivation, the cultures were centrifuged, and the ZEN degradation rate was tested. In the results (Figure 6), the supernatant had a higher degradation rate of 60.68%, indicating that the main degrading component was the supernatant of SY-3. The supernatant and cell pellets of SY-14 had higher degradation rates of 81.17% and 85.06%, respectively. The cell pellets and intracellular and cell walls of SY-20 had higher degradation rates of 30.14%, 70.06% and 30.77%, respectively.

## 3. Discussion

Some studies on the selection of strains with specific abilities or probiotic functions were obtained from the general environment, such as soil [24,25] and fermented food [18]. This method of selecting strains had no specificity, a wide range, or high randomness. Lee et al. [26] obtained a strain of *Bacillus amyloliquefaciens* from moldy corn samples, and almost completely removed ZEN (3.5 ppm) from the LB medium. According to the principle of enrichment cultures, specific culture conditions were provided to make specific microorganisms grow vigorously and achieve quantitative advantages, which was more conducive to the separation of specific microorganisms. This method of selecting specific functional strains could save time and improve the selection efficiency. We speculated that strains grown in moldy environments for a long time would more easily eliminate ZEN. Some studies have demonstrated that strains with ZEN removed were screened from the animal intestinal environment [21,22,27].

The purpose of this experiment was to obtain ZEN-degrading strains from pigs. Thus, in order to improve the probability of screening ZEN-degrading strains, a ZEN toxicological model of pigs was artificially created in this experiment. The intestinal flora of animals is affected by ZEN [28,29,30]. In our study, ZEN was added to the diet of pigs in order place the pigs in a state of toxicological challenge during the experimental period, and to improve the tolerance of pig intestinal flora to ZEN. The results showed that three out of twenty-five pig feces samples showed significant ZEN degradation abilities (>98%), as determined by HPLC. SY-3 was slightly more potent than SY-14 and SY-20, reaching 99.48%. The identification process was based on morphological, biochemical characterization, and phylogenetic analysis. The strain SY-3 was identified as *Proteus mirabilis*, and the two strains SY-14 and SY-20 were identified as *Bacillus subtilis*. The results demonstrated that the ZEN degradation ability of the strains was approximately 10 μg/mL under these experimental conditions. The ZEN degradation ability of the experimental results was higher than the ZEN degradation of some studies of primary screening results [19,31,32]. Using the ZEN toxicological model of pigs to select specific strains appears to be a viable option.

Many strains of *Proteus mirabilis* have been isolated from a wide variety of environments, but there are no reports on the degradation of ZEN by *Proteus mirabilis*. In this study, the degradation rate of ZEN reached 99.48% using *Proteus mirabilis* SY-3. *Proteus mirabilis* was first isolated from laying hens with salpingitis by Bisgaard [33]. In China, *Proteus mirabilis* was first isolated from dead broilers by Jiang et al. [34]. *Proteus mirabilis* is a Gram-negative bacterium that exists widely in natural, human and animal intestines, animal feces and clinical samples. *Proteus mirabilis* is a conditional pathogen [35]. Coker et al. [36] reported that the virulence factors of *Proteus mirabilis* included fimbriae, flagella, outer membrane protein, urease and hemolysin production, cell invasiveness, and iron acquisition. Whether *Proteus mirabilis* can become a strained resource beneficial to the development of the animal husbandry economy should be studied further.

Many studies have reported that microorganisms in the environment, animal feces, soils, and rumen fluid have been selected to degrade ZEN. Y-33 of *Bacillus subtilis* showed the capability to degrade ZEN, which was chosen from soils and water. The *Bacillus subtilis* was cultured with ZEN for 72 h in deMan Rogosa Sharpe (MRS) broth medium, and the ZEN degradation rate reached 78.3% [37].Cho et al. [38] proved that *Bacillus subtilis* degraded more than 95% of ZEN (0.25 mg/kg) in solid-state fermentation after 48 h, and degraded 99% of ZEN (1 mg/kg) in liquid medium after 24 h. Ju et al. [39] proved that *Bacillus subtilis* cultured with ZEN in the liquid medium after 24 h had a residual content of ZEN of 2.3%, and these authors also concluded that *Bacillus subtilis* had the strongest degradation effect in corn flour, and that the degradation rate was 75%. Chen et al. [40] argued that the ability of *Bacillus subtilis* to reduce the amount of ZEN reached 56% when cultured with the contaminated maize (5 mg/kg).

In our study, *Bacillus subtilis* SY-14 and SY-20 were isolated from pig feces, and the percentages of ZEN degradation of these two strains were 98.78% and 98.97%, respectively. Compared with other studies, the three strains in this study had higher degradation efficiencies of ZEN, but further studies on these strains are needed in order to improve the ability to degrade ZEN by optimizing the fermentation conditions. *Bacillus subtilis* has been used for a wide variety of applications, especially agricultural applications. *Bacillus subtilis* could also be used to improve the quality of crops, to prompt growth of crops, and to regulate soil nutrients [41]. *Bacillus subtilis* could be produced as a microbial preparation and used in the field of animal husbandry. Chang et al. [42] reported that *Bacillus subtilis* could be used as the main component of compound probiotics; if these are added to feed, they could increase the production performance and alleviate the hepatic tissue damage of broilers by aflatoxins B1 and ZEN. Zhang et al. [43] proved that *Bacillus subtilis*, if added to feed, could ease the toxicity of female pigs and alleviate cell damage. Shen et al. [44] demonstrated that biodegradable *Bacillus subtilis,* as an additive, could significantly lower the concentrations of immunoglobulin G (IgG), immunoglobulin M (IgM), interleukin-2 (IL-2) and tumor necrosis factor-α (TNF-α) of gilts which are fed a ZEN-contaminated diet. In our study, two Bacillus subtilis strains, SY-14 and SY-20, were isolated from pigs. These strains are easier to plant in the intestinal tract of pigs in theory, but the specific implantation methods need to be further studied.

There were several factors influencing the microbial degradation of compounds, such as the culture time, the culture temperature, the inoculation of the strain, and the pH value of the culture medium. Thus, the optimal conditions of ZEN degradation for the three strains should be studied carefully. Figure 5a shows the effect of different times on ZEN degradation activity in a culture. The ZEN-degrading rate remained stable after some time; this conclusion was consistent with the results of Tan et al. [45]. We can infer from the growth curve of the strain that as the culture time increased, the number of strains increased in the exponential phase, and the substances secreted by the strains that can remove ZEN were increased. When the strain was in the stationary phase, the quantity of strain remained static, and the concentration of substances attained the maximum. With increasing time, the strain was in the decline phase, and the content of ZEN remained unchanged. Some of the removed ZEN could be adsorbed by cells, and when the cells died, they lost the ability to adsorb ZEN, increasing the ZEN content in the culture medium.

Microorganisms are greatly affected by temperature, and a temperature that is too high or too low could affect the growth of strains. Microorganisms can cause the thermal inactivation of cell enzymes in a high-temperature environment. The respiratory enzymes are deformed when the temperature rises, resulting in a decrease in the bacterial activity. At low temperatures, microorganisms can reduce or stop their metabolism. The water in the protoplasm of the cell could freeze when the temperature is very low, leading to cell death [46]. The optimal temperature of the three strains was 37 °C, and the temperature was close to the optimum temperature of the enzyme reaction in animals.

Different inoculations also affected the degradation of ZEN. Less inoculation resulted in the prolongation of the lag phase of the strain, the substances of removed ZEN were produced slowly, and the degradation time of ZEN was prolonged. Nutrients were consumed rapidly when the inoculation was too high, later-growing strains were nourished, and ZEN was removed slowly. Different strains had different inoculations. Duan et al. [47] reported that the optimal inoculation of *Bacillus amyloliquefaciens* was 2%. In our study, when the inoculation of SY-3 was 3%, the degradation rate of ZEN was the highest. There was no significant difference when the inoculation of SY-14 was between 1% and 9%, but inoculation with 1% had a higher degradation rate of ZEN. When the inoculation of SY-20 was 1%, the degradation rate of ZEN was the highest.

A suitable pH value was beneficial to the growth of the strain. The culturing of the strain in an over-acidic or over-alkaline environment could affect the degradation rate of ZEN. Different strains had different optimal pH values to degrade ZEN. Tan et al. [45] reported that the optimal pH value of *Pseudomonas otitidis* for the biodegradation of ZEN was 4.5. In this study, the initial pH values of 9, 5–9 and 8 were the best optimal pH value ranges for SY-3, Sy-14 and SY-20, respectively.

The methods of the microbial removal of ZEN included mainly the following two aspects. The first was the absorption of ZEN by microorganisms. The second was that the structure of ZEN was destroyed by extracellular substances, and intracellular substances were secreted by the microorganisms and produced nontoxic products. El-Nezami et al. [48] studied the degradation mechanism of ZEN by *Lactobacillus rhamnosus*. ZEN was not detected in the culture supernatant but was recovered from the strain cells. The bacterial cells—after heating treatment—could still remove ZEN, so we inferred that the detoxification mechanism of *Lactobacillus rhamnosus* to ZEN was adsorption. Hsu et al. [49] showed that ZEN could be removed by *Bacillus licheniformis* cells, and the content of ZEN removed by heating and acid treatment was almost the same as the content of ZEN removed by living cells. *Bacillus licheniformis* was indicated to be able to remove ZEN by adsorption.

Figure 6 shows the effect of different treatments on the ZEN degradation activity in the culture. The supernatant and cells of the strain could remove ZEN. The further treatment of the cell suspension showed that the degradation of ZEN in the intracellular solution was lower than the degradation of ZEN in the cell wall (*p* < 0.05). We inferred that the main degrading component of SY-3 was the supernatant. There was no effect from the supernatant of SY-14 to degrade ZEN. The further treatment of the cell suspension showed that the degradation rate of ZEN by the intracellular fluid was close to the degradation rate of ZEN of the living strain (*p* > 0.05), such that we concluded that the main degradation component of SY-14 was intracellular fluid. The ability of SY-20-inactivated bacteria to remove ZEN was close to half the ability of SY-20-inactivated bacteria to remove the ZEN of the living strain (*p* < 0.05). The supernatant of SY-20 had a low degradation of ZEN. The further treatment of the cell suspension showed that the degradation of ZEN by intracellular fluid was higher than the degradation of ZEN by the cell wall (*p* < 0.05); thus, we inferred that the main degradation components of SY-20 were intracellular substances and the cell wall. In this study, the ability of the living strain to degrade ZEN was higher than the ability of the inactivated strain to degrade ZEN. After treatment with heating, the pooled active heat-inactivated culture retained a low ZEN degradation activity, which indicated that the section of degradation matter was inactivated by heating. Therefore, we could infer that the substance inactivated by heating was a protein structure.

Many studies have reported ZEN degradation mechanisms in microorganisms. Yiannikoris et al. [50] studied *Saccharomyces cerevisiae* with ZEN adsorption capacity for ZEN elimination mechanisms, and they identified that the critical mechanism is the β-D-glucan of the cell wall. The specific mechanism of ZEN degradation by the three strains in the study was unclear, and should be studied further. At present, an increasing number of studies have focused on the isolation of ZEN-degrading bacteria and their metabolites. Xiang et al. [51] reported that ZHD (an enzyme gene) was added to wort (the ZEN content was 10 μg/mL), and the degradation rate of ZEN could reach 90% after 12 min. Swamy et al. [52] showed yeast cell wall extract added to the diet could effectively reduce the toxic effect of ZEN on piglets. The above research showed that ZEN degradation enzymes and bacterial extracts have good application prospects in the animal husbandry and food industries. Therefore, the ZEN degradation mechanism of these strains from this experiment should be researched in depth.

## 4. Conclusions

In this study, three strains that degraded ZEN in LB medium from the feces of pigs were successfully isolated. Several factors affect the degradation capability of ZEN, including the optimal time, temperature, inoculation and pH value. The optimal time, temperature, inoculation and pH value of *Proteus mirabilis* SY-3 for the biodegradation of ZEN were 60 h, 37 °C, 3% and 9, respectively. The main degrading component was the supernatant. No other *Proteus mirabilis* strain has previously been reported to be capable of degrading ZEN. The optimal time, temperature, inoculation and pH value of *Bacillus subtilis* SY-14 for the biodegradation of ZEN were 36 h, 37 °C, 1% and 5–9, respectively. The main degradation component was intracellular fluid. The optimal time, temperature, inoculation and pH value of *Bacillus subtilis* SY-20 for the biodegradation of ZEN were 36 h, 37 °C, 1% and 8, respectively. The main degradation components were intracellular fluid and the cell wall.

## 5. Materials and Methods

### 5.1. Chemicals and Medium

ZEN, purchased from Sigma-Aldrich (St. Louis, MO, USA) and dissolved in methanol (1 mg/mL), was used as a standard stock solution in this study. The ZEN solution was stored in darkness at −20 °C. The methanol and acetonitrile were High Performance Liquid Chromatography (HPLC) grade, and were obtained from Shandong Yuwang Industrial (Shandong, China). The LB medium (pH 7.0) contained the following constituents: yeast powder 5 g/L, NaCl 10 g/L and tryptone 10 g/L. LB medium was autoclaved at 121 °C for 15 min. For the plate cultures, the previous medium solidified with 1.5% (*w*/*v*) agar was chosen.

### 5.2. ZEN-Challenged Pig Model

The protocols used in this experiment were approved by the Northeast Agricultural University Institutional Animal Care and Use Committee. All the Animal care and treatment complied with the standards described in the “Laboratory Animal Management Regulations” (revised 2016) of Heilongjiang Province, China. In this study, 6 healthy piglets (Duroc × Landrace × Yorkshire) with an average initial body weight of 9.92 ± 1.38 kg were randomly allocated into one group, and the group consisted of six replicates with one piglet per replicate. All of the pigs were individually penned in metabolism cages. Water and feed were provided ad libitum to the pigs. The pre-experiment lasted for 7 days. All of the pigs with an average body weight of 12.27 ± 1.78 kg were weighed at the end of the pre-experiment. The experiment lasted 20 days. The experimental diets were formulated to fit the nutrient requirements recommended by the NRC [53]. The ingredient composition and nutrient level of the basal diet are shown in Table 2. During the experimental period, 2 mg ZEN was added to the basal diet per kg in order to establish a model of ZEN toxicosis (the original ZEN content of the basal diet was 0.09 mg/kg). After the experiment, the feces of the pigs were collected in 5 mL cryogenic vials, mixed in equal volumes with glycerol–water solution, and stored at −80 °C.

### 5.3. Isolation of the ZEN-Degrading Bacteria

A portion (1 g) of each fecal sample was added individually to a 50-mL centrifuge tube with sterilized PBS (phosphate buffer saline). The centrifuge tube was shaken for 5 min. Subsequently, the tube was centrifuged for 5 min at 3000 rpm. The supernatants (10% inoculation) were added to LB medium (with 10 μg/mL ZEN), and were then incubated at 37 °C for 48 h in a rotary shaker (220 rpm). The above steps were repeated, and the supernatants (10% inoculation) obtained in the previous step were added to LB medium (with 15 μg/mL ZEN) and then incubated at 37 °C for 48 h in a rotary shaker (220 rpm). The final bacterial suspensions were obtained by an enrichment culture, and were then plated on sterilized LB agar plates. Colonies for further study were selected based on the morphological differences, and were inoculated in LB medium. A single bacterium was obtained after the above steps were repeated five times. The bacterial solution was mixed with a 60% sterilized glycerol–water solution and stored at −80 °C.

### 5.4. Screening for ZEN-Degrading Bacteria

A single bacterium was activated in a sterilized LB medium. The bacterial solution was adjusted to optical density (OD) value of 1 at 600 nm (OD_600_ = 1). The culture condition of a single bacterium was inoculation with 1% LB medium, while the ZEN concentration was 10 μg/mL. The positive controls contained no bacteria, and the negative controls for each strain contained no ZEN. The mixtures were incubated at 37 °C for 48 h in a rotary shaker (220 rpm). Three times, each sample was measured in parallel, and the degradation rate was analyzed by HPLC. By the end of the culture, an equal volume of methanol was added to the solution [24], and the mixture was whirled for 1 min at room temperature for more than 1 h. The methanol extracts of the culture were centrifuged for 10 min at 10,000 rpm. The supernatants were filtered through an organic filter (0.22 μm) and placed in vials. The ZEN was separated on a C18 column (4.6 mm × 250 mm; article size, 5 μm; Agilent Technology Inc, Santa Clara, CA, USA) with a mobile phase (*v*/*v*) of acetonitrile, methanol and water (4:23:23) at a flow rate of 1 mL/min, and was detected by a fluorescence detector at 274 nm and 440 nm. The assay temperature was 25 °C, with an injection volume of 10 μL. The relative reduction of ZEN in each suspension was calculated by the following equation: (1 − peak area of ZEN in the supernatant/peak area of ZEN in the positive control) × 100.

### 5.5. Morphological, Physiological, and Biochemical Characterization

The cell morphology was recorded by Gram staining. In order to investigate the physiological and biochemical characteristics, the standard techniques reported by Dong et al. [54] were performed, including the use of an oxidizing enzyme, catalase, methyl red, V-P, starch hydrolyzation, indole, glucose fermentation and fermentation of carbohydrate and alcohol (include: fructose, galactose, cellobiose, mannose, saccharose, lactose, maltose, and mannitol).

### 5.6. Phylogenetic Analysis

The 16S ribosomal DNA (rDNA) gene sequences of the selected strains were analyzed using universal primers 27F (AGAGTTTGATCMTGGCTCAG) and 1492R (TACGGYTACCTTGTTACGACTT). The bacterial solution was dissolved in a 50 μL Takara Lysis Buffer for Microorganisms to Direct PCR (Takara Biomedical Technology Co., Ltd., Beijing, China), and was centrifuged after denaturation. The supernatants were used as template DNA at 80 °C for 15 min. Next, 2×TransTaq^®^High Fidelity (HiFi) PCR SuperMix Ⅰ (TransGen Biotech, Beijing, China) was used for the PCR amplification. The PCR conditions were as follows: 94 °C for 5 min, 1 cycle; 95 °C for 10 s, 55 °C for 20 s, and 72 °C for 1.5 min, 35 cycles; and 72 °C for 10 min, 1 cycle. The 16S rDNA gene sequences obtained were analyzed through a BLAST search in the NCBI database. Phylogenetic trees were constructed by the MEGA 6.0 program using neighbor-joining.

### 5.7. Effect of the Culture Conditions on the Degradation of ZEN

The strains selected in the previous experiments for their high degradation rates were used in this experiment. The selected strains were prepared according to the previous methods described in the selection of ZEN-degrading bacteria. The bacterial solution was adjusted to OD_600_ = 1. A single bacterium was inoculated at 1% confluence in LB medium and medium at an initial pH of 7.0, while the ZEN concentration was 10 μg/mL. The mixtures were shaker-incubated (220 rpm) at 37 °C for 12 h, 24 h, 36 h, 48 h, 60 h and 72 h. The mixtures with 10 μg/mL ZEN were shaker incubated (220 rpm) for 48 h. The culture temperatures were modified by Zhao et al. [18], and were set at 20 °C, 25 °C, 30 °C, 37 °C, and 42 °C. Single bacteria were inoculated at 1%, 3.0%, 5.0%, 7.0%, and 9.0% in LB medium and medium at an initial pH of 7.0. The mixtures with 10 μg/mL ZEN were shaker-incubated (220 rpm) at 37 °C for 48 h. The single bacterium was inoculated at 1% in LB medium, and the medium’s initial pH was changed to 4.0, 5.0, 6.0, 7.0, 8.0, 9.0, and 10.0. The mixtures with 10 μg/mL ZEN were shaker-incubated (220 rpm) at 37 °C for 48 h. Three times, each sample was measured in parallel, and the degradation rate was analyzed by HPLC. The sample treatment method was mentioned in Section 5.4.

### 5.8. Determination of the Degradation Ability of the Strain Components in ZEN

A single bacterium was cultured in sterilized LB medium. The bacterial solution was adjusted to OD_600_ = 1, and was divided into three parts. A portion was autoclaved at 121 °C for 20 min. A portion was kept as it was. A portion was centrifuged for 10 min at 8000× *g*. The supernatant and cells were separated, and then the supernatant was filtered with a 0.22 μm filter before use. The pellets were washed twice in PBS and then given an equal volume of PBS. The supernatant and sediment were separated after the ultrasonic extraction of the mixture. The supernatant was filtered with a 0.22-μm filter before use. The sediment was washed twice with PBS, and then given an equal volume of PBS. The above six groups of samples with 5 μg/mL ZEN were incubated at 37 °C for 48 h in a rotary shaker (220 rpm). By the end of the culture, an equal volume of methanol was added to the solution, the mixture was whirled for 1 min, and it was then stood at room temperature for more than 1 h. The methanol extracts of the culture were centrifuged for 10 min at 10,000 rpm. The sample treatment method was mentioned in Section 5.4.

### 5.9. Data Analysis

The data were analyzed with Statistical Product and Service Solutions (SPSS 22.0) software (International Business Machines Corporation, Armonk, NY, USA). One-way ANOVA was used to compare the dataset in combination with Duncan’s multiple range test in order to determine the significant differences (*p* < 0.05) among the means. All of the results are presented as the mean ± standard deviation of three replicates.

## Figures and Tables

**Table 1:** The physiological and biochemical characteristics of strains SY-3, SY-14 and SY-20.

Characteristics	SY-3	SY-14	SY-20
Oxidase test	−	+	+
Catalase test	+	+	+
Glucose	+	+	+
Fructose	+	+	+
Galactose	+	−	−
Cellobiose	−	+	+
Mannose	−	+	+
Inulin	+	+	+
Saccharose	−	+	+
Lactose	−	+	+
Maltose	−	+	+
Mannitol	−	+	+
Methyl red test	−	+	+
Voges-Proskauer	−	+	+
Amylase	−	+	+
Indole	+	−	−

Key: “+”, positive; “−”, negative.

**Table 2:** Ingredient composition and nutritional level of the basal diet on an as-fed basis.

Ingredients	Content (%)	Nutritional Level	
Corn	60.60	Digestible energy (MJ/kg)	14.71
Full-fat expanded soybean	10.00	Crude protein (%)	19.47
Peeled soybean meal	15.00	Lysine (%)	1.41
Soybean protein concentrate	3.00	Calcium (%)	0.71
Fish meal	4.00	Total phosphorus (%)	0.60
Whole milk powder	2.00	Available phosphorus (%)	0.36
Soybean oil	2.00		
Lysine (79.8%)	0.24		
Methionine (98%)	0.04		
Threonine (98%)	0.08		
Calcium hydrogen phosphate	0.65		
Limestone	0.79		
Salt	0.40		
Choline chloride (30%)	0.20		
Premix ^1^	1.00		

^1^ Provided the following per kilogram of diet: Cu, 20.2 mg; Zn, 106.5 mg; Se, 0.3 mg; Mn, 1.03 mg; Fe, 120 mg; I, 0.2 mg; vitamin A, 5000 IU; vitamin D3, 1250 IU; vitamin E, 47.5 IU; vitamin K, 2.2 mg; vitamin B1, 3.6 mg; vitamin B2, 8.0 mg; vitamin B6, 4.1 mg; vitamin B12, 0.04 mg; pantothenic acid, 18 mg; niacin, 29.7 mg; folate, 1.9 mg; and biotin, 0.4 mg.

## Data Availability

The raw reads of the 16S rRNA sequencing were submitted to the GenBank databases under accession number MZ348886. The data presented in this study are available in the present article, and are shared with consent and in accordance with all of the authors.
